# Role of the Social Support and Health Status in Living Arrangement Preference of the Elderly in China—A Cross-Sectional Study

**DOI:** 10.3389/fpubh.2022.860974

**Published:** 2022-07-12

**Authors:** Longyan Cui, Jingshan Li, Danni Xie, Minghui Wang, Fanrong He, Junfeng Chen, Ding Ding

**Affiliations:** College of Public Health, Dalian Medical University, Dalian, China

**Keywords:** elderly, social support, health status, living arrangement preference, factors, CLHLS

## Abstract

**Background:**

Living arrangement of the elderly is one of the most important components that affect their quality of life in later years. The aging, with the phenomenon of low fertility rate and family structure transformation, has caused changes in the living arrangements of the elderly. This research aimed to find the elderly's living arrangement preferences and influencing factors.

**Methods:**

The data were obtained from The Chinese Longitudinal Healthy Longevity Survey (CLHLS) in the 2018, and the sample was comprised of 9,638 individuals aged ≥ 60 years. Independent variables were divided into social support, health status and so-economic status. Chi-square test and binary logistic regression were used to analyze the relationship between the above variates and living arrangement preferences.

**Results:**

Currently, in terms of living arrangement preferences, nearly half (45.6%) of the respondents choose not to live with their children. The binary model results showed that elderly who were married (OR = 0.166, 95% CI: 0.147–0.187), experienced more than 6 years of education (OR = 0.600, 95% CI: 0.517–0.695), ability of daily living (ADL) impaired (OR = 0.810, 95% CI: 0.720–0.912), suffering from multiple chronic diseases (OR = 0.803, 95% CI: 0.720–0.912), and obtained community services (OR = 0.884, 95% CI: 0.803–0.972) incline to not live with their children. The elderly who living in rural areas (OR = 1.244, 95% CI: 1.129–1.371), with an income of more than 500,000 yuan per year (OR = 1.557, 95% CI: 1.380–1.757), having children visiting regularly (OR = 1.405, 95% CI: 1.161–1.707) and receiving children's financial support (OR = 1.194, 95% CI: 1.080–1.319) are more likely to choose to live with their children.

**Conclusions:**

This study found that the living arrangement preferences of the elderly were affected by social support and health status, and living with children is no longer the only option for the elderly these days. The elderly care services provided by communities or professional care institutions may become the mainstream of taking care of the elderly citizens in the aging society. Improving the types and forms of community nursing services to increase the accessibility of these services; setting up elderly care institutions reasonably and equipping adequate professional nursing staff should be considered as priority measures.

## Introduction

Aging has become an issue that countries all over the world have to face. The United Nations predicted that one in five people will be aged 65 or older in 2050 (1). The astonishing proportion of older persons will not only bring formidable challenges to improve and maintain health systems and quality of life, but also change the dynamics of living arrangements in dramatic ways (2). Nowadays, various policies and measures to alleviate the challenges posed by aging society are being explored by many countries. Particularly, the theory of healthy aging with living arrangements as one of the components proposed by WHO, provides a theoretical basis and guidance for many countries (3, 4).

Living arrangements are defined by the composition or number of families and the identity of co-residents, and the types mainly include living with children, living with other relatives, living alone, and pension institutions (5). Globally, the proportion of elderly living alone or with only a spouse is increasing, while fewer are living with extended families. For example, living with at least one child or extended family member is the most common living arrangement for elderly in Africa, Asia, Latin America and the Caribbean. While, in Europe, North America, Australia and New Zealand, living with a spouse only was the most common living arrangement, followed by living alone (6). In general, traditional Chinese elderly are expected to live with family members, especially with their children. In fact, around 60% of the people aged 65 or above live with their children (7). However, according to the 6th Chinese Census in 2010, the elderly living alone or with their spouse accounted for more than 50% of the total elderly population; this number (the 5th Census was 38% in 2000) has risen nearly 12% in just 10 years (8). The empirical numbers of this investigation indicated that the traditional living arrangement has been transformed. The factors, including social economic development, urbanization, and the one-child policy in China, etc., are the main contributor of the rapid increase in the number of elderly households living alone or only with their spouses (9–11). Nowadays, the total number of elderly aged 60 and above in mainland China in 2020 is 264 million, accounting for 18.7% of the total population, subsequently, those who are living alone (or empty nesters) will increase to around 118 million.^1^ The rapid increase in the elderly will push the society to meet the challenge of providing more suitable elderly care and medical resources for the elderly. In this sense, the living arrangement of the elderly has been put a top priority of this study.

Considerable research on the living arrangements of the elderly has confirmed a series of determinants in the past decades, such as, death of spouse or other family members, economic environment, health or functional status, availability of children or relatives, etc. (12, 13). However, previous studies have mostly focused on the actual living arrangements of the elderly, and there were fewer studies on their living arrangement preferences (14). In recent years, A few studies have revealed that in developed countries, living alone or with a spouse only meets the desire for most people to live independently; in some developing countries, more and more elderly choose to live separately from their children (15). For instance, Yang's research has confirmed that independent living has become the mainstream of living arrangements for the elderly in China (16). Furthermore, studies from Asia have confirmed that the heterogeneity of the cultural basis of the living arrangements of the elderly. While, the existing considerable researches and theories supporting this field of research were based on data collected from Western countries, thus, the evidence from these studies has limitations on the fitness of non-Western societies (12).

Therefore, this study uses survey data on the Chinese Longitudinal Healthy Longevity Survey database to evaluate the relationship between individual health status, social support, personal socio-economic characteristics and the elderly's living arrangement preference. Our research mainly contributes to the relevant literature in two aspects. Firstly, this study describes the characteristics of China's housing model through the choice of the elderly's housing preferences, and fills in the gaps in the existing literature. Secondly, this research explores the relationship between living arrangements preferences and personal health, economic characteristics, and social support behaviors, providing empirical evidence for the implementation of relevant pension policies, and also providing references for other countries facing similar situations.

## Methods

### Data

**Figure 1:**
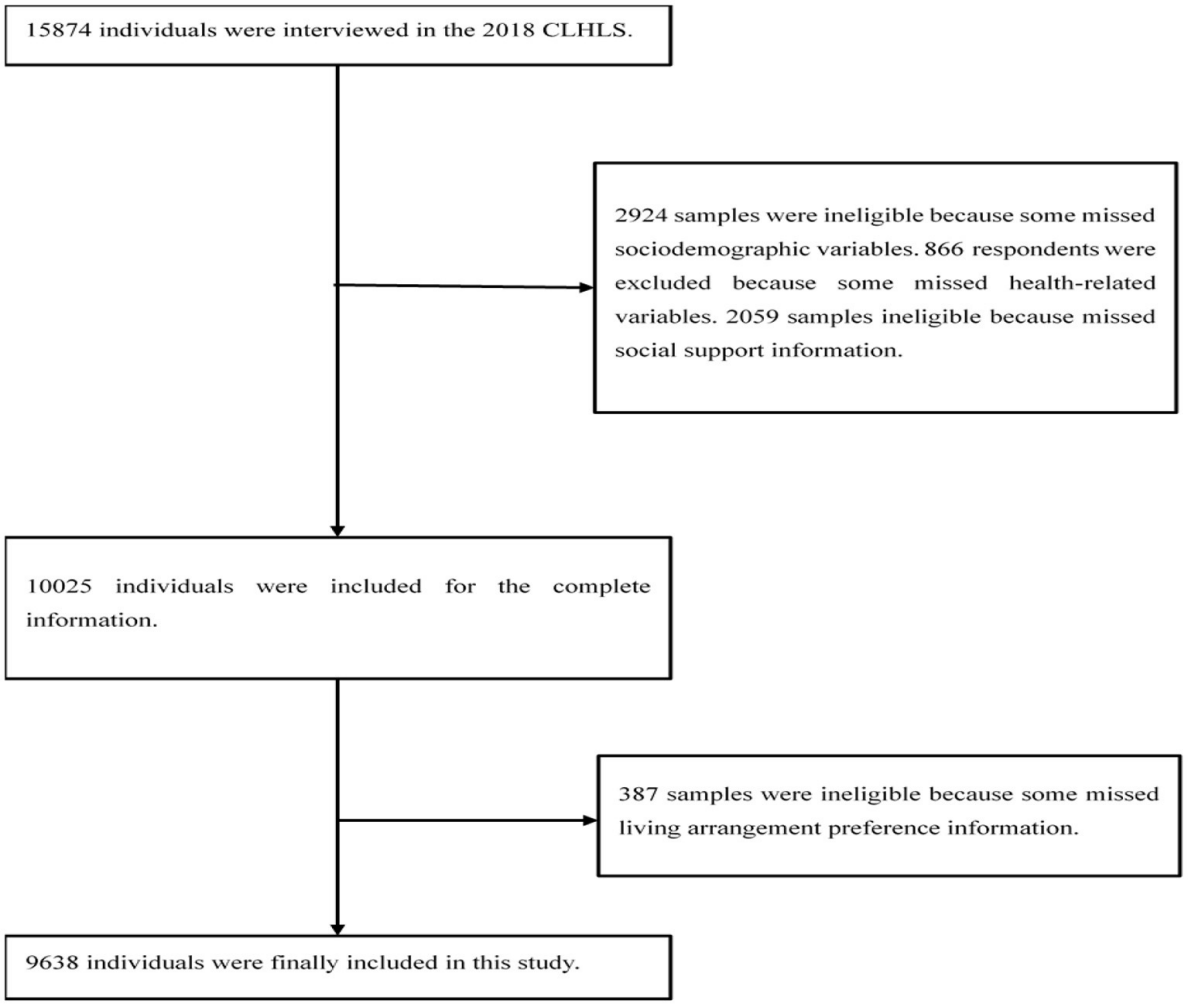
Participants' flow in the research.

The data were derived from the Chinese Longitudinal Healthy Longevity Survey (CLHLS) approved by Duke University and Peking University, which is mainly to understand the health status of the Chinese elderly population. All participants signed an informed consent (17). The survey covered 23 provinces out of 31 provinces in China, and the total population of the covered area accounts for about 85% of the all population of the country, which has a good national representativeness.^2^ The data can be obtained through the Open Research Data Platform of Peking University (http://opendata.pku.edu.cn/). This study selected the latest survey data (*N* = 15,874) released in 2018. The rules for the inclusion of research subjects were as follows: Firstly, those with incomplete basic information will be eliminated; secondly, those with insufficient health status and social support information will be ignored; thirdly, missing information about preference for living arrangements will be excluded; Finally, the sample size of this study was 9,638 (Figure 1).

### Variable

#### Dependent Variable

In this study, the item “Which kind of living arrangement do you prefer?” was used as the basis for evaluating the willingness of living arrangement. The answer options for this item mainly included the following five items: living alone (or with a spouse), no matter how far away the children live; living alone (or with a spouse), but preferably living nearby; living with children; old hospital, etc.; and I don't know. In addition to the choice of living with children, we have divided the other types of living preferences into not living with children (including living alone, institutional pension, etc.), that was (0 = not living with children, 1 = living with children).

#### Independent Variable

The human-environment theory indicated that individuals often try to maximize the coordination between the environment and needs by changing the environment or changing his/her perception of needs (12, 18). As this ability declines, personal behavior, especially that of elderly, is more affected by external environmental factors (19, 20). Previous considerable studies have also confirmed the impact of health status and family support on the living arrangements of the elderly (13, 21).

In terms of sociodemographic characteristics, gender (1 = male, 2 = female); age (60–69, 70–79, ≥80); education level (1 = 0 years, 1 = 1–6 years; 3 = 6+years) (22); residence (1 = city/town, 2 = rural); annual family income: (1 = 0–49,999 RMB, 2 = 50,000–99,999 RMB, 3 = ≥100,000 RMB); current residence mode (1 = with family, 2 = live alone, 3 = nursing home); marital status (1 = married, 2 = unmarried, including widowed, divorced, never married, etc.).

Social support is usually defined as “support that an individual obtains through social connections with other individuals, groups, and the larger community.” It is usually divided into emotional support (referring to provide care, empathy, trust and love) and instrumental support (tangible goods, services or assistance) (23). In terms of social support, outside and family-level variables were considered. For the social support obtained at the external level, the items in the questionnaire are mainly selected related to: Do you currently have older pension insurance?/Do you currently have medical insurance? (1 = Yes, 0 = No). In addition, what social services has your community provided to you? (There are 8 types of services, including personal daily care services, home visits, psychological consulting, daily shopping, social and recreation activities, human rights consulting services, health education, neighboring relations. As long as one answer is yes, it means that you have enjoyed community services). For the support received from the family, the questionnaire items are selected: In the past year, have you received financial support from your children and grandchildren (1 = Yes, 0 = No); Did your children come to visit you? (1 = Yes, 0 = No).

Health status mainly involves self-reported health, ability of daily living (ADL) and chronic diseases (24). Self-reported health is evaluated based on the personal health status of the elderly interviewed. There were five options: very good, good, fair, poor, and very poor. We define “very good” or “good” as “good” according to the response of the respondent; “normal”; and “poor” or “very bad” as “poor”. ADL disability (lack of the ability to perform daily activities) was described as difficulties related to six items: dressing, bathing, eating, going to bed or getting up, going to the toilet, and controlling thoughts. If one of the items cannot be completed independently, it is judged as disabled (0 is defined as disability, 1 = disability). We assessed the number of chronic diseases by asking each respondent to select from a list of 25 chronic diseases (e.g., Hypertension, Diabetes, Heart disease, Stroke, cerebrovascular disease, Bronchitis, emphysema, asthma, pneumonia, Pulmonary tuberculosis, Cataracts, Glaucoma, Cancer, Prostate tumor, Gastric or duodenal ulcer, etc.). It was expressed as (1 = no disease, 2 = one disease, 3 = two or more diseases).

### Analysis

The data were expressed as a percentage of the classification value. Firstly, the chi-square test was used to explore the differences between the living arrangement preferences of the elderly with different characteristics. Secondly, binary logistic regression was used to analyze and determine the relationship between social support, health status, and living arrangement preferences. According to the results of univariate analysis, gender, education level, age, marital, residence, current residence mode, and income were used as the benchmark model 1. Then, on the basis of model 1, ADL and Chronic diseases were put into Model 2. Next, based on Model 1, community services, children's financial support, children's visit were added in the Model 3. Finally, the variables included in the health status and social support were put into Model 1 to explore influencing factors of living arrangement preference. Data were expressed as OR and 95% CI. The test level was 0.05, and the *p*-value ≤ 0.05 was considered statistically significant. All statistical analyses were performed using SPSS25.

## Result

### The Characteristic of the Samples

The overall respondents, 4,244 (44.0%) were male, 4,658 (48.3%) have never been educated; 62.8% of the elder were 80 years old and above. The elderly in marriage accounted for 47.9% of the total; the proportion of elderly with an annual family income of <50,000 yuan has reached 57.0%. More than half of the elderly (96.4%) live with family members. There were differences in the living arrangement preferences of the elder with different gender, age, education level, residence, income, marital and current living mode (*p* < 0.005). Specifically, in terms of preference for living arrangements, the elderly who are female, uneducated, 80+, live in city/town, unmarried, have a family income of <50,000 yuan and live with family members, were more inclined to with children (Table 1).

**Table 1:** Respondents' characteristics and living arrangements preferences (*N* = 9,638).

**Variables**	**Total**	**Living arrangement preference**		**χ^2^**	***P***
	***N* (%)**	**With children** ***n* (%)**	**Not with children** ***n* (%)**		
**Gender**				200.100	<0.001
Male	4,244 (44.0)	1,964 (37.5)	2,280 (51.8)		
Female	5,394 (56.0)	3,276 (62.5)	2,118 (48.2)		
**Education**				422.048	<0.001
0	4,658 (48.3)	2,975 (56.8)	1,683 (38.3)		
1–6	3,063 (31.8)	1,559 (29.8)	1,504 (34.2)		
6+	1,917 (19.9)	706 (13.4)	1,211 (27.5)		
**Age**				664.708	<0.001
60–69	1,241 (12.9)	482 (9.2)	759 (17.2)		
70–79	2,345 (24.3)	859 (16.4)	1,486 (33.8)		
80+	6,052 (62.8)	3,899 (74.4)	2,153 (49.0)		
**Residence**				22.213	<0.001
City/town	5,507 (57.1)	2,880 (55.0)	2,927 (59.7)		
Rural	4,131 (42.9)	2,360 (45.0)	1,771 (40.3)		
**Marital status**				1,629.796	<0.001
Marriage	4,617 (47.9)	1,524 (29.1)	3,093 (70.3)		
Not marriage	5,021 (52.1)	3,716 (70.9)	1,305 (29.7)		
**Annual income**				63.507	<0.001
0–49,999	5,495 (57.0)	2,700 (61.4)	2,795 (53.3)		
50,000–99,999	2,064 (21.4)	854 (19.4)	1,210 (23.1)		
100,000~	2,079 (21.6)	844 (19.2)	1,235 (23.6)		
**Current living arrangement**				166.702	<0.001
With household member(s)	9,286 (96.4)	5,163 (98.5)	4,123 (93.7)		
Alone	320 (3.3)	61 (1.2)	259 (5.9)		
Nursing home	32 (0.3)	16 (0.3)	16 (0.4)		

*Living with children refers to the elderly living together with children. Not living with children means that the elderly only live with their spouses or in nursing institutions, welfare homes, etc*.

### Preference for Living Arrangements of the Elderly With Different Health Conditions and Social Support

The univariate analysis revealed that five variables were related to the preference of the elderly in living arrangements (Table 2). Specifically, compared with the elderly who don't live with their children, the elderly with impaired ability of daily living choose to live with their children at a higher rate, which was 33.9%. In addition, 31.3% of the elderly suffering from one chronic disease and 36.8% with two chronic diseases chose to live with their children. Besides, the proportion of elderly who received community services, financial support, and child visitation was more likely to live with them. Chi-Square χ^2^ test show that the ability of daily living, chronic diseases, community services, children's financial support, and children's visits were all significantly related to the living preferences of the elderly.

**Table 2:** Chi-Square analysis of the living arrangement preference of the elderly with different characteristics.

**Variables**	**Total**	**Living arrangement preference**		**χ^2^**	***P***
	***N* (%)**	**With children** ***n* (%)**	**Not with children** ***n* (%)**		
**Health condition**					
**Self-reported health**				2.909	0.234
Good	4,255 (43.8)	2,327 (44.4)	1,898 (43.2)		
Normal	4,183 (43.4)	2,233 (42.6)	1,950 (44.3)		
Poor	1,230 (13.8)	680 (13.0)	550 (12.5)		
**ADL disability**				200.935	<0.001
Yes	2,691 (27.9)	1,774 (33.9)	917 (20.9)		
No	6,947 (72.1)	3,466 (66.1)	3,481 (79.1)		
**Chronic diseases**				65.258	<0.001
0	2,787 (28.9)	1,668 (31.8)	1,119 (25.4)		
1	2,984 (31.0)	1,642 (31.3)	1,342 (30.5)		
2+	1,937 (40.1)	1,930 (36.9)	1,937 (44.1)		
**Social support**					
Older insurance				0.283	0.597
Yes	2,611 (27.1)	1,408 (26.9)	3,822 (27.4)		
No	7,027 (72.9)	3,832 (73.1)	3,195 (72.6)		
**Health insurance**				0.645	0.422
Yes	7,970 (82.7)	4,348 (83.0)	3,622 (82.4)		
No	1,668 (17.3)	892 (17.0)	776 (17.6)		
**Community services**					
Yes	6,206 (64.4)	3,305 (63.1)	2,901 (66.0)	8.706	0.003
No	3,432 (35.6)	1,935 (36.9)	1,497 (34.0)		
**Children's financial support**				13.649	<0.001
Yes	6,663 (69.1)	3,706 (70.7)	2,957 (67.2)		
No	2,975 (30.9)	1,534 (29.3)	1,441 (32.8)		
**Children visit**				16.600	<0.001
Yes	9,050 (93.9)	4,968 (94.8)	4,082 (92.8)		
No	588 (6.1)	272 (5.2)	316 (7.2)		

### Analysis on the Influencing Factors of the Elderly's Living Arrangement Preference

In the binary regression model, the meaningful variables in the univariate analysis were incorporated into the model to further explore the factors affecting the life preferences of the elderly. The results of Model 4 revealed that the elderly who married (OR = 0.166, 95% CI: 0.147–0.187), experienced more than 6 years of education (OR = 0.600, 95% CI: 0.517–0.695), ADL disabled (OR = 0.810, 95% CI: 0.720–0.912), multiple chronic diseases (OR = 0.803, 95% CI: 0.720–0.912) and have community services (OR = 0.884, 95% CI: 0.803–0.972) were more likely to not live with their children. Conversely, living in rural (OR = 1.244, 95% CI: 1.129–1.371), with an income of more than 50,000 yuan (OR = 1.557, 95% CI: 1.380–1.757), with children visiting regularly (OR = 1.405, 95% CI: 1.161–1.707) and children's financial support (OR = 1.194, 95% CI: 1.080–1.319) were more likely to choose to live with their children (Table 3).

**Table 3:** The binary logistic regression analysis on the living arrangement preference of the elderly [OR (95% CI)].

**Variable**	**Reference**	**Model 1**	**Model 2**	**Model 3**	**Model 4**
		**OR (95% CI)**	**OR (95% CI)**	**OR (95% CI)**	**OR (95% CI)**
**Gender**					
Female	Male	1.004 (0.905–1.113)	1.018 (0.918–1.129)	0.997 (0.899–1.106)	1.010 (0.910–1.121)
**Education** (**years)**					
1–6	0	0.968 (0.862–1.087)	0.964 (0.858–1.084)	0.963 (0.857–1.081)	0.960 (0.854–1.079)
6+		0.582 (0.502–0.673)***	0.591 (0.510–0.685)***	0.590 (0.510–0.684)***	0.600 (0.517–0.695)***
**Age**					
70–79	60-69	0.771 (0.663–0.897)**	0.784 (0.674–0.912)**	0.757 (0.651–0.881)***	0.770 (0.661–0.896)**
≥80		0.946 (0.812–1.103)	1.009 (0.863–1.179)	0.935 (0.802–1.091)	0.994 (0.850–1.162)
**Residence**					
Rural	City/town	1.275 (1.158–1.405)***	1.253 (1.137–1.381)***	1.265 (1.148–1.393)***	1.244 (1.129–1.371)***
**Marital status**					
Marriage	Not marriage	0.173 (0.154–0.194)***	0.167 (0.148–0.188)***	0.172 (0.153–0.193)***	0.166 (0.147–0.187)***
**Annual income**					
50,000–99,999	0–49,999	1.538 (1.364–1.733)***	1.551 (1.375–1.749)***	1.545 (1.370–1.743)***	1.557 (1.380–1.757)***
100,000~		1.862 (1.646–2.108)***	1.902 (1.679–2.155)***	1.899 (1.676–2.151)***	1.934 (1.706–2.193)***
**Current living model**					
Alone	With household member(s)	0.094 (0.070–0.126)***	0.089 (0.066–0.120)***	0.095 (0.071–0.127)***	0.090 (0.067–0.122)***
In a nursing home		0.447 (0.211–0.948)*	0.471 (0.221–0.999)*	0.494 (0.231–1.053)	0.517 (0.242–1.107)
**ADL disability**					
Yes	No	—	0.798 (0.709–0.898)***	—	0.810 (0.720–0.912)**
**Chronic diseases**					
1	0	—	0.887 (0.788–0.999)*	—	0.891 (0.791–1.004)
2+		—	0.798 (0.712–0.894)***	—	0.803 (0.717–0.900)***
**Community services**					
Yes	No	—	—	0.872 (0.792–0.959)**	0.884 (0.803–0.972)*
**Children's financial support**					
Yes	No	—	—	1.207 (1.092–1.333)***	1.194 (1.080–1.319)**
**Children visit**					
Yes	No	—	—	1.407 (1.163–1.702)***	1.405 (1.161–1.707)***

*p < 0.05; **p < 0.01; ***p < 0.000.

*Model 1 only includes individual socioeconomic variables such as gender, age, education level, residence, annual income, marital status, and current residence mode*.

Model 2, based on Model 1, the variables of personal health status, including ADL and chronic diseases were included.

*Model 3, based on Model 1, community services, children's financial support, children's visits were included*.

*Model 4 includes variables such as basic personal information, health status, and social support*.

## Discussion

As a traditional country with family culture at its core, it is customary for Chinese seniors to live with their children when they are old in order to enjoy their twilight years. While this research has demonstrated that in the preference of living arrangements, the proportion of elderly who tend to live with their children (54.4%) and the proportion who choose not to live with their children (45.6%) (including only living with their spouse, nursing care institution, etc.) are roughly close, which is contrary to the traditional model. Similarly, the research by Qu also has revealed that the independence of Chinese elderly living is increasing, and the willingness to live with their children has shown a downward trend (25). The above-discovered trends have indicated that the traditional lifestyle with children is not as mainstream choice as before.

The reasons for this change in the elderly's live arrangements may be as follows: The evidence from China's 2018 edition of the Blue Book of Social Integration of the Floating Population Report showed that the China's floating population reached 245 million, accounting for 18% of China's total population in 2016. The post-80s floating population is the main body whose proportion is about 65% (26). The rapid socio-economic development has prompted young people to move to urban areas or cities with better urban economic development to find jobs and a better life, which makes the elderly have to live separately from their children (10, 27). Besides, the Chinese government has also promulgated a series of policies, such as community care; integrated medical care; smart pension; and long-term pension insurance. Moreover, it's also actively setting up considerable elderly care institutions and accelerating the training of professional nursing staff. These measures have broadened the forms and channels of care, and the elderly are no longer limited to living with their children in order to obtain necessary life care.

In addition, not only China, but other countries are also facing such a change. Previous researches have also confirmed that in developed and some developing countries, the elderly are more willing to choose to live separately from their children (15, 28). Hence, in the context of severe global aging, it's important for all countries to foresee the changes in the living arrangements of the elderly.

### Social Support With Living Arrangement Preference

This study indicated that the elderly who have received community services tend not to live with their children. Generally, the types of community services mainly include life support, medical services, spiritual comfort and other services. It's a consensus that family medical services in China have become more convenient for the elderly. When the elderly don't live with their children, some basic services to maintain their daily lives could be provided by the community, which can avoid the adverse effects due to lack of care from family members. Usually, family health services are mainly provided by community health service centers (29). Studies have shown that the high availability of community family health services provides the elderly with basic nursing services and meets their needs (30). In addition, some health policies provided by the community have indeed improved the convenience and success rate of medical treatment, and improved people's health (31, 32). These favorable conditions guarantee basic medical needs for the elderly, thus they could choose not to live with their children.

Research also suggested that the preference of the elderly who have children to provide financial support, children to visit regularly, or live in rural areas are more likely to live with their children. Firstly, influenced by the traditional Chinese Confucian culture, it's customary for the youngster to care and support the older family members, especially their patents. The degree of this concept is even more profound (7, 23). Secondly, while health-related resources and pension resources in rural areas are scarce, increasing age leads to the decline of personal physical functions, and the elderly still need to rely on the economic and daily help provided by their children to maintain their life (33). Besides, by obtaining financial and emotional support from their children, the elderly not only have better financial ability to obtain health-related resources to maintain healthy, but also the opportunity to actually live in the caring and loving family as a whole. In such a more harmonious family environment, the elderly will be more willing to live with their children. Apart from getting family care for the elderly themselves, they could also be of great help within their capacity, such as looking after grandchildren, cooking and cleaning etc. (28).

### Health Status With Living Arrangement Preference

In terms of living arrangements and health status, this study has provided evidence that elderly with multiple physical disabilities and impaired activities of daily living prefer not to live with their children. Similarly, research on the elderly in Japan has also found that the deterioration of health conditions has increased the possibility that the elderly in Japan to switch from living with their children to living with their spouse only or living alone (12). In a traditional family-oriented culture, for many Chinese elderly, the social network of the elderly is family-centric, and children play an important role, which is their important spiritual support (6, 34). Nevertheless, this type of living arrangement preference isn't permanent. As proposed by Zhou, Z, the poor health will indeed lead to changes in living arrangements (35). With deteriorating health conditions, elderly will be needing more specialized care or treatment from professional medical institutions. The current medical-care which integrates elderly care model provided by relevant medical institutions or nursing homes meets the needs of these elderly. Furthermore, as the body function declines, elderly will inevitably lead to soaring demand for long-term care (4).

For Chinese elderly, long-term care is mainly provided through informal care arrangements, for instance co-residence with their children (36, 37). However, youngster nowadays tend to be turning away from their hometown to seek better lives in big cities like Beijing, Shanghai or Shenzhen; and all of that resulted in the insufficiency of free family care (27, 38). Moreover, the number of community service agencies and facilities in China has shown an overall upward trend. The coverage of comprehensive service facilities in urban communities was 92.9%, and 59.3% in rural communities (39). Furthermore, 38,000 elderly care institutions and 8.238 million elderly care service beds can be provided in 2020 (40). Hence, when the physical condition of the elderly gradually deteriorates, the preference of housing arrangements may gradually shift to nursing institutions. On one hand, shifting to nursing institutions create a considerable relief of burden for the children, on the other hand, living in nursing facilities could guarantee their medical needs, hence, ensure their healthiness.

### Other Factors

Factors such as being married, 6 years of education and above, and family income above 50,000 yuan per year were also found to be related to the preference of the elderly's living arrangements. Usually, those who live with their spouse are better able to cope with poor health and maintain their current living arrangements (41). What's more, the elderly with higher socioeconomic status choose not to live with their children, which can avoid potential intergenerational conflicts with the family and enjoy better quality of life (10). In addition, research has revealed that home care in China is increasingly expanding to elderly parents who cannot provide care but are able to purchase it. Such elders usually want to avoid causing trouble to their children and seek better institutional care than can be provided at home (42). Finally, for the elderly with higher family income, a better economic foundation provides a strong guarantee for them to obtain relevant health resources, which in turn makes their health conditions better. People usually think that adult will take care of their elderly parents, but in reality, parents actually provide more help than they get (43). Therefore, the healthy elder were more likely to live with their children in order to provide some assistance in daily life (28).

There are some strengths and limitations in the present study. The advantage of this study is that it examines the living arrangement preferences and influencing factors of the elderly in China from the perspective of individual subjective willingness, which has reference significance for countries that are also facing the dilemma of aging. However, constrained by the CLHLS data structure, we failed to provide more detailed information about community services, social support, living arrangement preferences in the elderly. Moreover, the cross-sectional data were insufficient to demonstrate causality. Subsequent research can further analyze the living arrangement preferences of the elderly on this basis.

## Conclusion

This research has demonstrated that social support and health status play an important role in the living arrangement preferences of the elderly. Since the impact of the transformation of family structure, social culture, and socio-economic development, the mode of living for the elderly has gradually shifted from living with children to autonomous home-based elderly care, which means that the living arrangements for the elderly will be less dependent on their children. Following are some suggestions that can be adopted. Firstly, advocate the intergenerational living model of “divide without separation”. By learning from the housing preferential policies of Singapore, Japan and other countries, encourage children or family members to live near the elderly, continue to play an important role in informal support, and provide life care and spiritual comfort for the elderly; Secondly, improve developmental family support policies (e.g., establish a caregiver allowance system). Moderate inclusive benefits to solve various practical difficulties encountered by family members in the process of caring for the elderly, to ensure the sustainability of family care; Thirdly, with the help of modern advanced technology, build a smart elderly care service system, so as to provide personalized elderly care service packages for the elderly at home, such as daily life care, medical care services and other high-quality services.

## Data Availability Statement

The datasets presented in this study can be found in online repositories. The names of the repository/repositories and accession number(s) can be found at: http://opendata.pku.edu.cn/.

## Ethics Statement

The studies involving human participants were reviewed and approved by Center for Healthy Aging and Development Studies at Peking University and Duke University. The patients/participants provided their written informed consent to participate in this study. LC registered the platform and obtained permission to use the data.

## Author Contributions

LC mainly analyzed the data and wrote the manuscript. DD designed the study and reviewed the paper. JC provided advice on the writing of the paper. DX, MW, JL, and FH responsible for collecting and cleansing the data. All authors contributed to the article and approved the submitted version.

## Funding

This research has supported by major projects in Liaoning Province (L21CRK001), Research on Embedded Community Home-based Elderly Care Service Support Based on Elderly Care Needs of Low-income Families (L21ZD015), and Regional Health Performance Evaluation Theory in the Background of Implementing Healthy China Strategy and method research.

## Conflict of Interest

The authors declare that the research was conducted in the absence of any commercial or financial relationships that could be construed as a potential conflict of interest.

## Publisher's Note

All claims expressed in this article are solely those of the authors and do not necessarily represent those of their affiliated organizations, or those of the publisher, the editors and the reviewers. Any product that may be evaluated in this article, or claim that may be made by its manufacturer, is not guaranteed or endorsed by the publisher.

## Footnotes

^1^Available online at: http://www.stats.gov.cn/tjsj/zxfb/202105/t20210510_1817176.html (accessed December 11).

^2^Available online at: http://cnsda.ruc.edu.cn/index.php?id=52572397&r=projects/view (accessed December 11).
